# Global climate-change trends detected in indicators of ocean ecology

**DOI:** 10.1038/s41586-023-06321-z

**Published:** 2023-07-12

**Authors:** B. B. Cael, Kelsey Bisson, Emmanuel Boss, Stephanie Dutkiewicz, Stephanie Henson

**Affiliations:** 1 National Oceanography Centre, Southampton, UK; 2 Department of Botany and Plant Pathology, Oregon State University, Corvallis, OR USA; 3 University of Maine, Orono, ME USA; 4 Center for Global Change Science, Massachusetts Institute of Technology, Cambridge, MA USA

**Keywords:** Ocean sciences, Climate-change ecology

## Abstract

Strong natural variability has been thought to mask possible climate-change-driven trends in phytoplankton populations from Earth-observing satellites. More than 30 years of continuous data were thought to be needed to detect a trend driven by climate change^1^. Here we show that climate-change trends emerge more rapidly in ocean colour (remote-sensing reflectance, *R*_rs_), because *R*_rs_ is multivariate and some wavebands have low interannual variability. We analyse a 20-year *R*_rs_ time series from the Moderate Resolution Imaging Spectroradiometer (MODIS) aboard the Aqua satellite, and find significant trends in *R*_rs_ for 40% of the global surface ocean. The climate-change signal in *R*_rs_ emerges after 20 years in similar regions covering a similar fraction of the ocean in a state-of-the-art ecosystem model^2^, which suggests that our observed trends indicate shifts in ocean colour—and, by extension, in surface-ocean ecosystems—that are driven by climate change. On the whole, low-latitude oceans have become greener in the past 20 years.

## Abstract (web summary)

An analysis of satellite data from July 2002–June 2022 shows that ocean colour, or remote-sensing reflectance, changed significantly during this period, and that this trend is likely to be driven by climate change.

## Main

Climate change is causing alterations in marine ecosystems, and is expected to increasingly cause such changes in the future^3^. Surface-ocean ecosystems cover 70% of Earth’s surface and are responsible for approximately half of global primary production^4^. Such communities are known to be changing at specific locations for which long-term data are available^5,6^. Detecting climate-change-driven trends in ocean ecosystems on a global scale, however, is challenging because of the difficulties of making oceanographic measurements at sufficiently large spatial and long temporal scales.

Satellite remote sensing is the only means to obtain time series of marine ecosystems on a global scale, because it is the only way to obtain measurements at the required scales. Ocean-colour satellites, which measure the amount of light radiating from the ocean and atmosphere from Earth’s surface, have been collecting global measurements for decades. A great deal of research has focused on detecting long-term trends in ocean-colour data, particularly in chlorophyll *a* (Chl) and primary productivity over large regions^7–11^. However, several studies^1,2,12^ have found that more than 30 years of data are required to detect climate-change-driven trends in satellite-derived Chl (μg l^−1^), the most frequently used product derived from ocean colour, even on regional scales. Chl provides information on the abundance of phytoplankton (the photosynthesizing microscopic organisms in the ocean), and can be estimated from empirically derived ratios and/or differences of ocean-colour *R*_rs_ (ref. ^13^). Because no single satellite mission has lasted a sufficient duration, and the intercalibration of merged multi-satellite products for robust, quantitative trend detection is challenging^12,14–17^, it has not so far been possible to determine for a given location whether Chl is changing with climate. Advances in statistical methods have allowed the detection of trends in large-scale regional Chl averages^18^, but it is difficult to distinguish for a given location whether Chl is or is not changing, and to determine whether any trends can be attributed to climate change.

That said, the MODIS sensor aboard the Aqua satellite (hereafter, MODIS-Aqua) has far surpassed its originally planned mission duration of 6 years, having just completed 20 full years collecting high-quality global ocean-colour data. The key variable provided by MODIS-Aqua (and any ocean-colour sensor) is *R*_rs_, which is the ratio of water-leaving radiance to downward irradiance incident on the ocean surface. *R*_rs_ is derived from MODIS-Aqua measurements in several wavebands within the visible spectrum, from 412 nm in the blue part of the spectrum to 678 nm in the red. Similarly to Chl, *R*_rs_ is an indicator of the state of the surface-ocean microbial ecosystem; *R*_rs_ is therefore considered an ‘essential climate variable’ by the Global Climate Observing System. Again similarly to Chl, trends in *R*_rs_ are not trivial to interpret ecologically or biogeochemically^19–23^ (Supplementary Information), but do reflect changes in surface-ocean ecology. There are persistent uncertainties in converting *R*_rs_ to Chl and other ecosystem properties such as phytoplankton carbon. Nonetheless, as *R*_rs_ does encode combined information about surface ecosystems and dissolved and particulate organic matter, any trend in *R*_rs_ reveals notable changes in the components of surface-ocean ecology and biogeochemistry with optical signatures. Furthermore, any change in *R*_rs_ corresponds to changes in the light environment itself, which will affect phytoplankton and thus ultimately lead to ecosystem changes.

**Extended Data Fig. 1:**
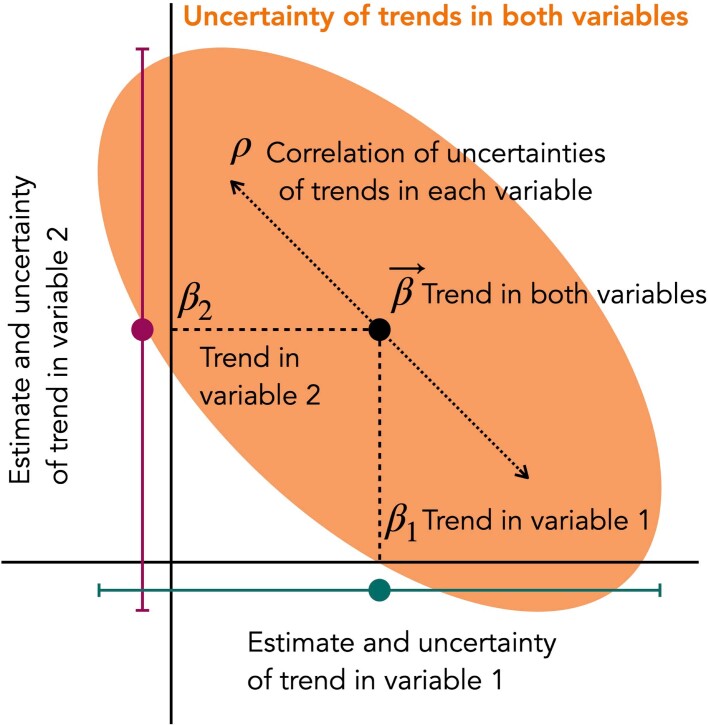
Schematic to illustrate multivariate trend detection. Here a two-dimensional case is shown. Teal and purple points and error bars indicate estimates and uncertainties (for example, 2 − *σ* if SNR ≥ 2 is of interest) of trends in two different variables, *β*_1_ and *β*_2_. Black point and orange ellipse indicate estimate and uncertainty in the two-dimensional variable **β** = (*β*_1_, *β*_2_). Dotted arrow indicates correlation (*ρ*) between uncertainties of estimates in each variable, which determines the angle made by the ellipse. In this graphical illustration, estimated trends in *β*_1_ and *β*_2_ are not significant, but the estimated trend in **β** is, because the orange ellipse does not contain the origin, but the purple and teal error bars cross through the *x*-axis and the *y*-axis, respectively.

Time-series data are the best way to identify long-term changes in an ecosystem^24^. Ocean-colour sensors are known to perform quite differently to each other—even copies of the same sensor on a different satellite platform^16^. Thus, the 20-year MODIS-Aqua record, as the longest single-sensor time series, constitutes a unique dataset. This dataset presents an opportunity to revisit the possibility of detecting trends in ocean colour from satellite data and attributing them to climate change. The principal reasons one might expect this to be possible are, first, that *R*_rs_ is multivariate, being measured by MODIS-Aqua at several wavebands, whereas Chl is univariate, meaning that *R*_rs_ potentially encapsulates a stronger signal than Chl (Extended Data Fig. 1); and, second, that some *R*_rs_ wavebands exhibit lower interannual variability than Chl (ref. ^2^), meaning that *R*_rs_ potentially has lower noise. In a model of complex global ocean ecosystems, climate-change-driven trends in *R*_rs_ have been shown to indicate changes in phytoplankton community structure and become distinguishable from natural variability more rapidly than trends in Chl (ref. ^2^). However, these multivariate advantages may not be sufficient to permit the detection of trends because *R*_rs_ is known to be strongly correlated between different wavebands^25^, reducing the effective dimension of the measurement^26^, and autocorrelation in *R*_rs_ may persist even at the annual timescale, reducing the effective sample size of a given *R*_rs_ time series. Solutions to both of these issues are possible, however. Multivariate regression allows the trends (and uncertainties in those trends) in multiple variables to be estimated simultaneously, while accounting for correlations between dependent variables^27^. Methods also exist to account for autocorrelation in regression analysis, such as the Cochrane–Orcutt procedure^28^, which estimates and subtracts the autoregressive component. In essence, then, such a regression maximizes the signal (number of simultaneous variables) used to detect a trend while also minimizing the noise (interannual variability in those variables) and accounting for correlations between variables and years.

### Observations

**Fig. 1:**
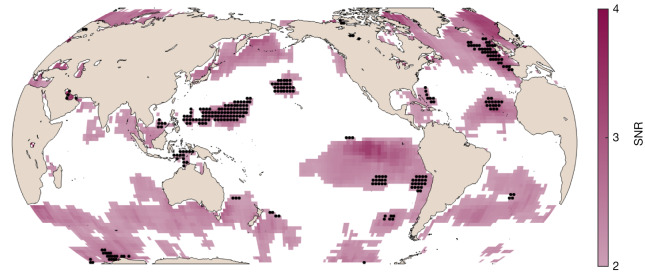
Map of locations where the ocean-colour trend SNR is higher than 2 for a 20-year annual time series. The intensity of the purple colour indicates the SNR. Black stippling indicates regions with significant trends in Chl as well (10% of the ocean). MODIS-Aqua data from July 2002 to June 2022.

To investigate possible trends in ocean colour, we performed such an autocorrelation-corrected multivariate regression on the first 20 years of MODIS-Aqua ocean *R*_rs_ data, spanning July 2002–June 2022 (Methods). We find significant trends, here defined as a signal-to-noise ratio (SNR) higher than two, in 40% of the ocean (Fig. 1; SNR > 2 corresponds to a confidence level around 95%). By contrast, only a small fraction of this portion of the ocean has significant trends in Chl (10%, black stippling in Fig. 1), such that even if the black stippled areas in Fig. 1 are excluded, 30% of the total ocean area has a significant trend in the *R*_rs_ product of ocean colour. These results are insensitive to significance level or spatial resolution (Methods).

We also note that these trends are not associated with changes in sea surface temperature (SST (°C)). When the same analysis is performed for MODIS-Aqua-based SST (Methods), we find significant *R*_rs_ trends in 36% of the ocean with a significant SST trend. Because 40% would be expected if *R*_rs_ trends were unrelated to SST trends, this suggests that the detected changes in *R*_rs_ are not related to changes in SST. Instead, changes in *R*_rs_ might be due to other drivers, such as changing mixed-layer depth or upper-ocean stratification^29^. These drivers are known to affect plankton community structure and biomass, and are expected to change with climate, but are more difficult to detect trends in over shorter time periods (that is, 20 years) than SST because they are measured less precisely.

**Extended Data Fig. 2:**
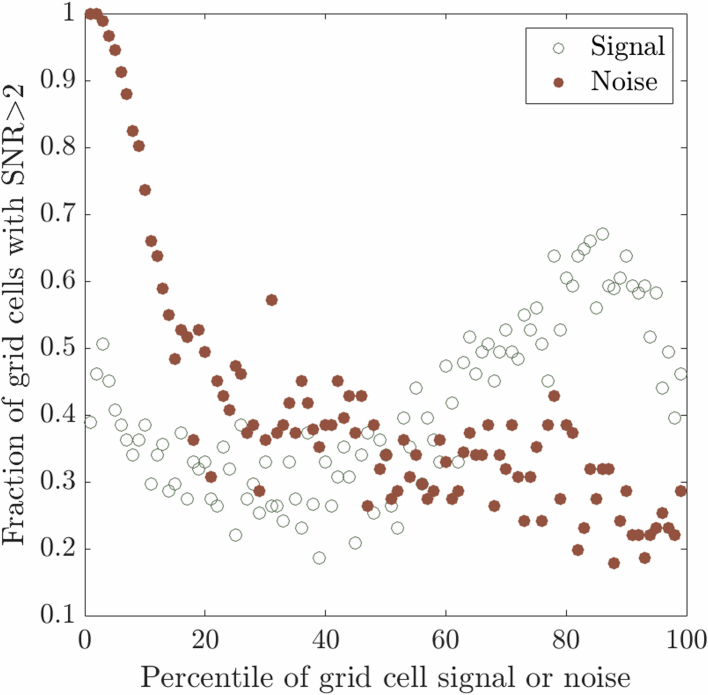
SNR > 2 regions are low noise, not high signal. Scatter plot of the fraction of grid cells with SNR > 2 versus the percentile of grid cell ‘signal’ (the magnitude of the trend, empty green points) and ‘noise’ (the uncertainty in the trend, filled orange points). Lower-‘noise’ regions more often have SNR > 2, whereas high-‘signal’ regions more often have SNR < 2, indicating that places with significant trends are those with the lowest trend uncertainty, owing to low interannual variability, rather than because they have the strongest trends.

**Extended Data Fig. 3:**
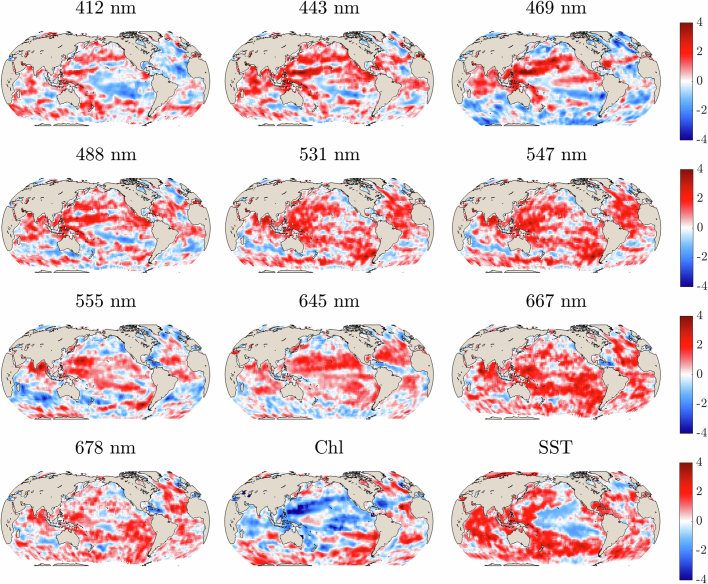
SNR changes are spectrally broad and variable. Maps of the SNR of univariate regressions of each wavelength, Chl and SST, with Cochrane–Orcutt procedure applied. Blue or red indicate a negative or a positive trend, respectively, and the intensity of colour indicates SNR.

**Extended Data Fig. 4:**
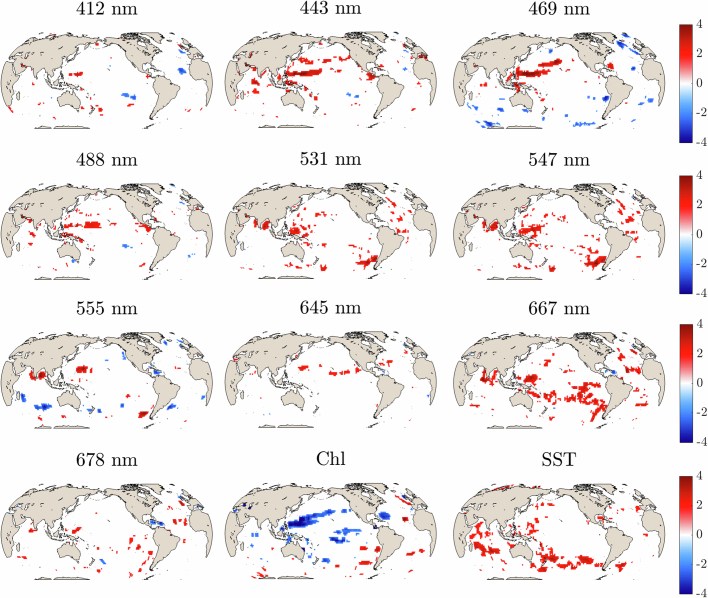
SNR > 2 changes are spectrally broad and variable. Same as Extended Data Fig. 3 but in which only locations with SNR > 2 are coloured.

We thus find that a vast swathe of the ocean has a significant trend in *R*_rs_, when considering many wavebands at the same time. Significant trends tend to occur in low-‘noise’ (that is, weak interannual variability) subtropical and tropical regions, rather than high-‘signal’ regions (Extended Data Fig. 2). The likelihood of SNR exceeding 2 and a trend being detectable increases markedly with decreasing noise levels, but does not consistently increase with increasing signal levels. Significant trends are also neither spectrally narrow (that is, linked to any particular waveband) nor spectrally flat (that is, lacking a spectral signature) (Extended Data Figs. 3 and 4).

### Model

**Fig. 2:**
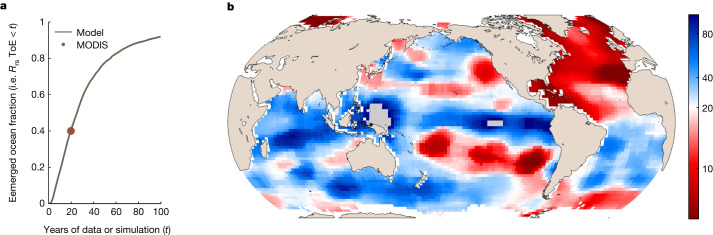
The modelled *R*_rs_ ToE of 20 years or less is similar to the location and extent of the observed 20-year data where SNR is higher than 2. **a**, Cumulative distribution function of the ToE of the ocean-colour trend in the model simulation. The orange point indicates the fraction of the total surface-ocean area with a significant trend in the 20-year MODIS-Aqua time series. Compare this with Fig. 10 in ref. ^2^, which shows less than 10% of the ocean with an emerged Chl trend after 20 years. **b**, Map of the ToE in the model simulation (median = 25 years). Grid cells are coloured by percentile, with white at 20 years, such that all white and red grid cells have a ToE of 20 years or less, and all blue grid cells have a ToE of more than 20 years. Grey grid cells do not have significant *R*_rs_ trends over the twenty-first century. See ref. ^2^ for a similar plot for Chl.

A key question is whether the identified trends are driven by climate change. To test this, we performed the same analysis on MODIS-like *R*_rs_ data simulated by a numerical model of a complex global ocean ecosystem and associated biogeochemical cycles^2,30^. The model simulates the changes to the marine ecosystem and optics over the course of the twenty-first century under a scenario of high greenhouse-gas emissions (Methods). By also considering a control simulation (that is, without perturbation from increased emissions), we can attribute changes to climate change. We analysed this model in terms of the time of emergence (ToE (years))^31^, which quantifies how long it takes for the climate-change-driven trend in a simulation with climate change (that is, a forced simulation) to emerge (with a SNR of 2) from the natural variability in a simulation without climate change (that is, a control simulation), both over the period 2000–2105. For the model *R*_rs_, the ToE is 20 years or less in 41% of the ocean, a comparable fraction to the 40% of the ocean for which we find a significant trend in MODIS-Aqua *R*_rs_ (Fig. 2a,b). The (area-weighted) median ToE across the entire model surface ocean is 25 years. By comparison, the ToE is 20 years or less for less than 10% of the ocean for Chl^2^, underscoring that climate-change-driven trends in *R*_rs_ can emerge much faster than those for Chl, and on a similar timescale to the observational period investigated here. Given the coarse resolution of the model, it only crudely captures some of the features of the physical circulation in the ocean, such as narrow current systems (for example, the Gulf Stream or equatorial currents). As such, direct comparisons of finer-scale features between model and satellite observations should be done with care. Nonetheless, similar broad regions in both cases are responsible for the significant trends after 20 years, notably the North Atlantic, the subtropical and subpolar North Pacific, the tropical and subtropical Eastern South Pacific, and the Indian and Atlantic longitudes of the Southern Ocean. Although this is, arguably, the only numerical model suitable for such investigations, which limits the strength of any attribution statement that can be made from it, the consistency in the overall extent and the general location of significant trends in the observations and emerged climate-change-driven trends in the model suggest that the observed trends are indeed driven by climate change. In the model, because changes in community structure emerge much faster than those of Chl or other optically relevant properties, the early emergence of *R*_rs_ trends is linked to phytoplankton community structure, which influences food webs, biogeochemical cycles and marine biodiversity.

### Discussion

Changes to the surface-ocean ecosystem will affect *R*_rs_ (see idealized examples provided in the Supplementary Information). From these considerations, the changes in *R*_rs_ and the spatial patterns seen in Extended Data Fig. 3 are complex, likely to be multifaceted and defy simple description. In the broadest terms, increases in *R*_rs_ are more frequent than decreases, and increasingly so for intermediate wavelengths, suggesting that the ocean is on the whole becoming greener. This greening could result for instance from an increase in detrital particles, which would increase backscattering at all wavelengths and absorption at shorter wavelengths. However, it could also result from other possible ecosystem shifts, such as a simultaneous increase in zooplankton and coloured dissolved material. Nonetheless, and regardless of any comparison with model trends, the observed changes in *R*_rs_ will necessarily have ecological implications. Irrespective of which optical constituent(s) in the surface ecosystem changed to produce a trend in *R*_rs_, any such optical change will alter the light environment. Because light is a key driver of phytoplankton communities, any change in the light environment—whether due to changes in in-water optical constituents or changes in light availability entering the ocean—will lead to a change in the surface-ocean ecosystem.

Altogether, these results suggest that the effects of climate change are already being felt in surface marine microbial ecosystems, but have not yet been detected because previous studies have considered Chl or other univariate approaches. *R*_rs_ facilitates the early detection of climate-change signals by integrating, and being sensitive to, changes in the properties of surface-ocean ecosystems. *R*_rs_, and thus surface-ocean ecology, has changed significantly over a large fraction of the ocean in the past 20 years. The changes in *R*_rs_ that we have identified have potential implications both for the role of plankton in marine biogeochemical cycles and thus ocean carbon storage, and for plankton consumption by higher trophic levels and thus fisheries. Our findings therefore might be of relevance for ocean conservation and governance. For instance, knowledge of where the surface-ocean microbial ecosystem is changing might be useful for identifying regions of the open ocean in which to establish marine protected areas under the United Nations high seas treaty on the biodiversity of areas beyond national jurisdiction. The identified locations with changes in *R*_rs_ are consistent with where changes are expected in drivers such as upper-ocean stratification, but might be more easily detectable on the global scale—as we have done here—thanks to the multivariate and low-interannual-variability nature of *R*_rs_. This highlights the value of long-term satellite missions like MODIS-Aqua and of space agencies maintaining missions for as long as is feasible. That significant trends occur primarily where interannual variability is low means that a similar signal may be expected to emerge in other portions of the ocean in coming years, although the MODIS-Aqua mission is scheduled to end in the near future. Thus for future work, merged multi-satellite products, as well as work that is currently underway to improve them, are essential. Ongoing work^32^ interpreting *R*_rs_ could shed light on what the trends found here indicate about precisely how surface-ocean ecology is changing^33,34^; we hope that the results presented here will spur further work to this end. Given the key role of plankton ecosystems in marine food webs, global biogeochemical cycles and carbon cycle–climate feedbacks, detecting change in these ecosystems is of great utility.

## Methods

We generated a 20-year annual time series of MODIS-Aqua *R*_rs_ and Chl by extracting the monthly level-3, 4-km *R*_rs_ and Chl values from July 2002 to June 2022 from https://oceancolor.gsfc.nasa.gov/l3/. We use the first 240 months of the standard monthly 9-km MODIS-Aqua 10 ocean wavebands of *R*_rs_, centred at 412 nm, 443 nm, 469 nm, 488 nm, 531 nm, 547 nm, 555 nm, 645 nm, 667 nm and 678 nm (https://modis.gsfc.nasa.gov/about/specifications.php). The 2022 reprocessing of *R*_rs_ and Chl was used, which reduces atmospheric correction errors and, crucially, minimizes any instrumental drift through updated sensor calibrations. Monthly data were aggregated into years each beginning in July, and data were averaged spatially to 2° resolution, resulting in a 90-by-180-by-20-by-10 array (respectively latitude, longitude, year and waveband), and a 90-by-180-by-20 array for Chl. Years beginning in July were used because the earliest MODIS-Aqua output available is from July 2002, so our dataset represents the first 20 years of MODIS-Aqua data. Regression is performed on annual data because performing a regression on monthly data would provide negligible benefit in terms of distinguishing a multidecadal trend, while coming at the cost of having to estimate additional parameters to represent the seasonal cycle and while imposing additional assumptions about the annual cycle. MODIS-Aqua was selected because it is now a 20-year record, the longest single-satellite *R*_rs_ product available at present. Merged products were not considered because although they incorporate additional data and reduce the risk of possible sensor degradation issues, there are known issues with satellite intercalibration that are challenging to deal with quantitatively in detecting significant trends over time^12,14–16^. MODIS-Aqua also provides a daytime SST (°C) product, for which we generated a comparable time series (that is, 20 July–June years at 2° spatial resolution).

For each 2°-by-2° grid cell, we then performed a multivariate regression of *R*_rs_ versus time. All analyses were performed in MATLAB 2021b. In essence, we calculate the trend, represented by a vector **b**, in the seven-dimensional *R*_rs_ space, while accounting for correlations between years and wavelengths. The uncertainties in the trends are the result of interannual variability, and are represented by a covariance matrix *C*. The off-diagonal elements of this matrix correspond to the covariance of uncertainties in the trends of different wavelengths, because if two wavelengths are correlated, the uncertainties in their trends will also be correlated. Before performing the regression, the serial autocorrelation in the signal was removed using the Cochrane–Orcutt procedure^28^. This works by iteratively estimating then subtracting the autocorrelated component of a signal until the autocorrelation is not statistically significant. For locations with significant autocorrelation (47% of grid cells), one iteration was applied, and then a second iteration was applied for grid cells whose autocorrelation continued to be significant (11% of grid cells). No more than two iterations were applied to any grid cell because 2% of grid cells had significant autocorrelation at the 5% level after the application of zero-to-two iterations. Our conclusions are not affected by this choice; for instance, applying one iteration to all grid cells equally yielded a negligible difference. The same approach is applied to the Chl time series. We then calculate the SNR in each case according to

$${\rm{SNR}}=\frac{\sqrt{{\sum }_{i}{b}_{i}^{2}}}{\sqrt{\frac{{\bf{b}}}{\sqrt{{\sum }_{i}{b}_{i}^{2}}}C(\frac{{\bf{b}}}{\sqrt{{\sum }_{i}{b}_{i}^{2}}}{)}^{{\prime} }}}$$

where **b** is the vector of trend estimates for each waveband and *C* is the variance-covariance matrix of **b**. In other words, SNR is the magnitude of the multivariate trend vector (see Extended Data Fig. 1), divided by the projection along this vector of the multivariate uncertainty of this multivariate trend. This is analogous to a *z*-score, or the number of standard uncertainties away from zero that a slope of a linear regression is in one-dimensional ordinary least squares regression. The only differences here are (i) we remove the autocorrelated component of each signal before performing the regression; and (ii) we have multiple dependent and correlated variables, so our trend is a vector rather than a scalar, and our uncertainty in that vector is a matrix owing to the correlations between the dependent variables, so we need to project that uncertainty matrix along that trend vector to get the ratio of the trend’s magnitude to its uncertainty.

For Chl, that is, the univariate case, this reduces to ${\rm{SNR}}=b/\sqrt{C}$, where *b* is the magnitude of the trend and *C* is the uncertainty of this trend. Note that uncertainty in these trends is effectively entirely due to interannual variability; a 2° × 2° annual measurement represents the aggregation of a vast amount of data, so by the law of large numbers there is negligible uncertainty in the sample average, and therefore trend uncertainty is dominated by interannual variability and the statistical method described above is justified. (For future work on small spatial scales, considering the uncertainty in the average of small numbers of data points might be important for robust uncertainty quantification). When computing fractions of the ocean with a significant trend, we account for the difference in surface area of different grid cells. We use the standard SNR = 2 as a threshold because this corresponds to a significance level of around 95% (strictly, 95.45%). Our conclusions are not sensitive to this choice: for a SNR ≥ 1.645, corresponding to a 90% confidence level, we find significant *R*_rs_ trends over 57% of the ocean (of which 19% has a Chl trend), whereas for a SNR = 2.576, corresponding to a 99% confidence level, we find significant *R*_rs_ trends over 20% of the ocean (of which 4% has a Chl trend). Note that our results are also not sensitive to the choice of spatial resolution; if we use a 1° or 4° resolution, we still find a significant *R*_rs_ (Chl) trend in 40% (10%) of the ocean using a SNR = 2 threshold. (We report all values to two significant digits because the third significant digit is resolution-dependent.) Similarly, our results with respect to SST are not sensitive to choice of SST product; when using the COBE-SST product^35^, we find the same lack of relatedness between SST and *R*_rs_ trends, with 36% of locations with significant SST trends having significant *R*_rs_ trends (40% expected if they are perfectly unrelated; cf. 36% with MODIS-Aqua SST).

For Extended Data Fig. 3 we performed the same procedure as above for each individual MODIS-Aqua waveband of *R*_rs_. Extended Data Fig. 4 is identical to Extended Data Fig. 3 but with locations where SNR < 2 for all wavebands removed, to show that individual wavebands have significant trends in small and overlapping regions, underscoring that the detected trends are due to the multivariate nature of *R*_rs_ and not associated with any individual waveband. We also performed this analysis for SST to compute the overlap between significant trends in *R*_rs_ and SST as described in the main text.

The biogeochemical model is the same as that used in a previous study^2^. Model output was taken from 10.7910/DVN/08OJUV. This is a complex ocean ecosystem and biogeochemistry model, resolving the major elemental cycles and eight types of phytoplankton. The ecosystem and biogeochemical cycles are forced with output from an earth system model of intermediate complexity^36^. From an 1860 spin-up, two simulations are performed: one is a control simulation run with constant 1860 concentrations of greenhouse gases, and a second is run with a high-emissions scenario with increasing concentrations of greenhouse gases (similar to Representative Concentration Pathway 8.5). Thus, the differences between the simulations indicate anthropogenically driven climate change. Each simulation is run for 250 years, nominally 1860 to 2110, and the analysis described here was performed on the last 106 years (that is, nominally from 2000 to 2105). The model resolves radiative transfer as described previously^30^ to generate *R*_rs_ at 25-nm resolution from 400–700 nm. We refer to previous work^2,30^ and references therein for further details and model validation. We linearly interpolate model *R*_rs_ to the MODIS-Aqua spectral waveband peaks (412, 443, 469, 488, 531, 547, 555, 645, 667 and 678 nm). Linearly interpolating the spectra to 1-nm resolution and convolving with the MODIS-Aqua spectral response functions did not affect the result. The model’s spatial resolution is 2° by 2.5° with 22 vertical layers. The ocean physics shows a realistic year-to-year variability in surface temperature and produces interannual variability (for example,the El Niño–Southern Oscillation) with frequency, seasonality, magnitude and patterns in general agreement with observations. Because of the high computational demand of this model, we use a single climate simulation from an ensemble of perturbed physics, perturbed initial conditions and varied emissions scenarios, with a medium effective climate sensitivity of approximately 3.0 °C (ref. ^36^). The control simulation showed that there were no significant drifts in the ecological or optical properties discussed here.

Using this model, we perform the same multivariate regression as above. Note that we perform this regression on the full model time series, rather than the first 20 years, because the utility of the model for our study is to test whether it is possible for climate-change-driven *R*_rs_ trends to emerge from interannual variability faster than Chl trends, and over a similar timescale to the period for which we have observations. We then calculate, following previous work^2^, the ToE for each grid cell according to ToE = 2 × (standard deviation)/(trend), where the standard deviation is that of the annual means at any grid location in the control run and the trend is that of the full forced simulation. Calculating and removing any drift in the control simulation negligibly affected this calculation.

### Reporting summary

Further information on research design is available in the Nature Portfolio Reporting Summary linked to this article.

## Online content

Any methods, additional references, Nature Portfolio reporting summaries, source data, extended data, supplementary information, acknowledgements, peer review information; details of author contributions and competing interests; and statements of data and code availability are available at 10.1038/s41586-023-06321-z.

## Supplementary information

Supplementary Information Appendix 1 - discussion of interpretations of trends in remote-sensing reflectance.

Reporting Summary

## Data Availability

Remote-sensing data are available from https://oceancolor.gsfc.nasa.gov/l3; the specific data product names are the first 240 months of the monthly 9-km standard MODIS-Aqua *R*_rs_ at 412, 443, 469, 488, 531, 547, 555, 645, 667, and 678 nm. Model outputs are available from 10.7910/DVN/08OJUV.

## Code availability

Code (in MATLAB 2021b) is available at 10.5281/zenodo.13736237 and https://github.com/bbcael/Rrs_trends.
