# Author Correction: Global climate-change trends detected in indicators of ocean ecology

**DOI:** 10.1038/s41586-024-08090-9

**Published:** 2024-10-24

**Authors:** B. B. Cael, Kelsey Bisson, Emmanuel Boss, Stephanie Dutkiewicz, Stephanie Henson

**Affiliations:** 1 National Oceanography Centre, Southampton, UK; 2 Department of Botany and Plant Pathology, Oregon State University, Corvallis, OR USA; 3 University of Maine, Orono, ME USA; 4 Center for Global Change Science, Massachusetts Institute of Technology, Cambridge, MA USA

**Keywords:** Ocean sciences, Climate-change ecology

Correction to: *Nature* 10.1038/s41586-023-06321-z Published online 12 July 2023

The original version of this article contained two errors in the analysis. The wrong elements of the covariance matrix of the regression coefficients were accidentally used for the signal-to-noise ratio calculations, and three ocean wavebands of MODIS-Aqua remote sensing reflectance were accidentally excluded. The correct version of the code is available at 10.5281/zenodo.13736237 and https://github.com/bbcael/Rrs_trends, as is the original version of the code which it replaces. After correcting these errors, the fraction of the ocean for which an ocean colour trend is detected is now 40% (previously 56%) at the 95% confidence level; a trend is now detected for 56% of the ocean at the 90% confidence level. The fraction of the ocean for which a modeled ocean colour trend emerges after 20 years is now 41% (previously 46%); the locations of these regions now more closely match where trends are detected in remote sensing reflectance. Other results are affected by this change negligibly or not at all. The consequent changes to Figs. 1,2 and Extended Data Figs. 2–4 and text have been corrected in both the PDF and HTML versions of the article. The Supplementary information file accompanying this notice shows the original and corrected figure files.

## Supplementary information

Original and corrected figures

