# Supplementary material for: Global climate-change trends detected in indicators of ocean ecology
Source: Nature. 2023 Jul 12;619(7970):551–4. doi: 10.1038/s41586-023-06321-z (PMC10356596; doi:10.1038/s41586-023-06321-z)
Supplement: Supplementary file 1 — Appendix 1 - discussion of interpretations of trends in remote-sensing reflectance. [file 41586_2023_6321_MOESM1_ESM.pdf]

nature portfolio

https://doi.org/10.1038/s41586-023-06321-z

Supplementary information

Global climate-change trends detected in indicators of ocean ecology

In the format provided by the authors and unedited

Appendix SI.1: Rrs trend interpretation

Interpreting multivariate trends in Rrs is fundamentally challenging due to the non-linear interactions among optical constituents in the water column. Rrs is proportional to total backscattering (b) divided by the sum of total absorption (a) and backscattering. The quantity and composition of dissolved and particulate material in the water column adjusts a and b differently [1]. For example, spectral absorption from phytoplankton varies with abundance (including pigment packaging effects, [2]), composition (including size, [3, 4, 5]), and physiology (including non photochemical quenching, [6]). At the same time, backscattering is affected by phytoplankton abundance [7], community composition (e.g., calcifying algae versus not, [8]), and even higher trophic levels (i.e., zooplankton, [9]). Thus, one cannot look at a single Rrs wavelength or even combination of Rrs wavelengths and rigorously attribute changes to alterations in any specific optical constituents. There are likely multiple combinations of changes to the different optical properties that would lead to the same Rrs change. However, one can speculate on possible explanation for trends based on what is known optically about different materials. Here we provide a thought experiment for the purposes of providing constraints on interpretation in future work.

If just colored dissolved matter (CDM) were to increase due to either enhanced cell stress and microbial remineralization of organic matter, absorption would increase especially in the blue wavelengths, causing lower Rrs at 412 and 443 but similar Rrs at wavelengths 469 nm and greater [10]. If just non-algal particles increased due to increased fecal production or sediment flux into the ocean, both absorption and backscattering would increase. The magnitude of absorption typically greatly exceeds that of backscattering, so Rrs changes would be more sensitive to the absorbing characteristics of the detritus. As with CDM, the wavelengths most affected are within the blue, but depending on the associated a and b changes, there could also be little Rrs change spectrally. If phytoplankton composition changed but all other attributes were held constant (i.e., physiology, biomass), a range of spectral Rrs changes are possible. For example, divinyl chl-a and divinyl chl-b are unique to *Prochloroccocus* [11], a cyanobacterium, and these two pigments have absorption peaks at ~450nm, ~475 nm, ~655, and ~670nm [12]. A shift to a community dominated by *Procholoroccus* (possible in regions of the ocean with reduced nutrient supply) would result in lower Rrs at 443nm, 469nm, and 667 nm in the satellite data. We note that within a cyanobacteria grouping, monovinyl chl-a is always present in *Synechococcus* or *Trichodesmium* yet this pigment is also shared across red and green algal lineages as well, making it non-diagnostic for detecting these other two cyanobacteria groups. Comparable complexities exist across all phytoplankton groups. A community shift to just diatoms would be characterized by absorption in pigments including diadinoxanthin, fucoxanthin, and monovinyl chl-a, which are always present, and may

also be characterized by absorption in others (e.g., zeathanthin, chlorophyll c1 + c2 and chlorophyll c3, which are also shared by dinoflagellates and crysophytes). Diadinoxanthin has absorption peaks at ~460nm and ~500nm and is low throughout the rest of the spectrum. Fucoxanthin has peaks at ~455nm and ~470nm, and monovinyl chl-a has peaks around ~440nm, ~490nm and ~660nm. Community composition aside, if phytoplankton chlorophyll increased but biomass stayed constant, backscattering would be primarily unchanged, leading to just increases in the absorption peaks of monovinyl chlorphyll-a, causing reduction in Rrs at 412, 443, 469, and 667nm. Increases in phytoplankton biomass would cause increases to both absorption and backscattering, but if both change by an order of magnitude, Rrs will be unchanged. If diel vertical migration were to shift such that more zooplankton were active in surface water during the day, we would expect higher backscattering and therefore higher Rrs, but the spectral dependencies are poorly known. However, we should emphasis that any of the idealized changes mentioned above would alter the spectral quality of the light environment. Give the key importance of spectral light to phytoplankton competitiveness, any change in light will feedback and impact the phytoplankton assemblage. Subtle shifts are altogether difficult to detect and interpret, especially without ancillary data or process modeling. We suggest that continued study and understanding of the changes in Rrs is crucial, especially given our finding that multivariate trend analysis using Rrs wavebands offer an earlier signal of changes than other ocean colour products.

References

[1] Howard R Gordon et al. “A semianalytic radiance model of ocean color”. In: *Journal of Geophysical Research: Atmospheres* 93.D9 (1988), pp. 10909–10924.

[2] Norman B Nelson, Barbara B Pr´ezelin, and Robert R Bidigare. “Phytoplankton light absorption and the package effect in California coastal waters”. In: *Marine Ecology Progress Series* (1993), pp. 217–227.

[3] Shovonlal Roy et al. “The global distribution of phytoplankton size spectrum and size classes from their light-absorption spectra derived from satellite data”. In: *Remote Sensing of Environment* 139 (2013), pp. 185–197.

[4] S Sathyendranath et al. “Remote sensing of phytoplankton pigments: a comparison of empirical and theoretical approaches”. In: International Journal of *Remote Sensing* 22.2-3 (2001), pp. 249–273.

[5] Alison P Chase et al. “Evaluation of diagnostic pigments to estimate phytoplankton size classes”. In: *Limnology and Oceanography*: *Methods* 18.10 (2020), pp. 570–584.

[6] Peng-Wang Zhai et al. “Radiative transfer modeling of phytoplankton fluorescence quenching processes”. In: *Remote sensing* 10.8 (2018), p. 1309.

[7] Michael J Behrenfeld et al. “Carbon-based ocean productivity and phytoplankton physiology from space”. In: *Global biogeochemical cycles* 19.1 (2005).

[8] WM Balch et al. “Surface biological, chemical, and optical properties of the Patagonian Shelf coccolithophore bloom, the brightest waters of the Great Calcite Belt”. In: *Limnology and Oceanography 59.5* (2014), pp. 1715– 1732.

[9] Michael J Behrenfeld et al. “Global satellite-observed daily vertical migrations of ocean animals”. In: *Nature 576.7786* (2019), pp. 257–261.

[10] Norman B Nelson and David A Siegel. “The global distribution and dynamics of chromophoric dissolved organic matter”. In: *Annual review of marine science 5* (2013), pp. 447–476.

[11] Suzanne Roy et al. Phytoplankton pigments: characterization, chemotaxonomy and applications in oceanography. Cambridge University Press, 2011.

[12] Annick Bricaud et al. “Natural variability of phytoplanktonic absorption in oceanic waters: Influence of the size structure of algal populations”. In: *Journal of Geophysical Research: Oceans* 109.C11 (2004).
