# Supplementary material for: Global climate-change trends detected in indicators of ocean ecology
Source: Nature. 2023 Jul 12;619(7970):551–4. doi: 10.1038/s41586-023-06321-z (PMC10356596; doi:10.1038/s41586-023-06321-z)
Supplement: Supplementary file 2 — Reporting Summary [file 41586_2023_6321_MOESM2_ESM.pdf]

nature portfolio

Corresponding author(s): B. B. Cael

Last updated by author(s): Jun 4, 2023

Reporting Summary

Nature Portfolio wishes to improve the reproducibility of the work that we publish. This form provides structure for consistency and transparency in reporting. For further information on Nature Portfolio policies, see our Editorial Policies and the Editorial Policy Checklist.

Statistics

For all statistical analyses, confirm that the following items are present in the figure legend, table legend, main text, or Methods section.

n/a	Confirmed	
☐	☒	The exact sample size (*n*) for each experimental group/condition, given as a discrete number and unit of measurement
☒	☐	A statement on whether measurements were taken from distinct samples or whether the same sample was measured repeatedly
☐	☒	The statistical test(s) used AND whether they are one- or two-sided<br>*Only common tests should be described solely by name; describe more complex techniques in the Methods section.*
☐	☒	A description of all covariates tested
☐	☒	A description of any assumptions or corrections, such as tests of normality and adjustment for multiple comparisons
☐	☒	A full description of the statistical parameters including central tendency (e.g. means) or other basic estimates (e.g. regression coefficient) AND variation (e.g. standard deviation) or associated estimates of uncertainty (e.g. confidence intervals)
☐	☒	For null hypothesis testing, the test statistic (e.g. *F*, *t*, *r*) with confidence intervals, effect sizes, degrees of freedom and *P* value noted<br>*Give P values as exact values whenever suitable.*
☒	☐	For Bayesian analysis, information on the choice of priors and Markov chain Monte Carlo settings
☒	☐	For hierarchical and complex designs, identification of the appropriate level for tests and full reporting of outcomes
☒	☐	Estimates of effect sizes (e.g. Cohen's *d*, Pearson's *r*), indicating how they were calculated

Our web collection on statistics for biologists contains articles on many of the points above.

Software and code

Policy information about availability of computer code

Data collection	No software was used for data collection.
Data analysis	Matlab 2021b was used for data analysis. Code is available at doi:10.5281/zenodo.4441150.

For manuscripts utilizing custom algorithms or software that are central to the research but not yet described in published literature, software must be made available to editors and reviewers. We strongly encourage code deposition in a community repository (e.g. GitHub). See the Nature Portfolio guidelines for submitting code & software for further information.

Data

Policy information about availability of data

All manuscripts must include a data availability statement. This statement should provide the following information, where applicable:

- Accession codes, unique identifiers, or web links for publicly available datasets
- A description of any restrictions on data availability
- For clinical datasets or third party data, please ensure that the statement adheres to our policy

Remote sensing data area available from https://oceancolor.gsfc.nasa.gov/l3; the specific data product names are the first 240 months of the Monthly 9km Standard MODIS-Aqua Remote sensing reflectance at 412/443/488/531/547/667/678nm. Model outputs are available from https://doi.org/10.7910/DVN/08OJUV.

Research involving human participants, their data, or biological material

Policy information about studies with human participants or human data. See also policy information about sex, gender (identity/presentation), and sexual orientation and race, ethnicity and racism.

Reporting on sex and gender	N/A
Reporting on race, ethnicity, or other socially relevant groupings	N/A
Population characteristics	N/A
Recruitment	N/A
Ethics oversight	N/A

Note that full information on the approval of the study protocol must also be provided in the manuscript.

Field-specific reporting

Please select the one below that is the best fit for your research. If you are not sure, read the appropriate sections before making your selection.

☐ Life sciences ☐ Behavioural & social sciences ☒ Ecological, evolutionary & environmental sciences

For a reference copy of the document with all sections, see nature.com/documents/nr-reporting-summary-flat.pdf

Ecological, evolutionary & environmental sciences study design

All studies must disclose on these points even when the disclosure is negative.

Study description	We analyzed the MODIS-Aqua satellite's remote sensing reflectance data for 20-year trends. We found significant trends over much of the ocean. The location and extent of these trends corresponds closely with the forced trends in the first 20-years of a simulation with a complex ecosystem model, indicating that these trends may be due to climate change.
Research sample	The data used here are remote sensing reflectance from NASA's MODIS-Aqua satellite, over the first 20 full years of its mission. These are chosen because this is the longest ocean color satellite mission and therefore most suitable for our research question investigating climatic trends.
Sampling strategy	Data were measured by NASA's MODIS-Aqua satellite.
Data collection	Data were taken from NASA's ocean color website and coarsened to 2° spatial resolution according to standard methods.
Timing and spatial scale	Data are global (over the ocean) in spatial extent and span from July 2002 – June 2022 at monthly resolution in time.
Data exclusions	No data were excluded.
Reproducibility	No experiments were conducted.
Randomization	Randomization was not relevant as data are spatially explicit and analyzed at 2° resolution.
Blinding	Blinding was not relevant as we were investigating trends in global satellite records.

Did the study involve field work? ☐ Yes ☒ No

Reporting for specific materials, systems and methods

We require information from authors about some types of materials, experimental systems and methods used in many studies. Here, indicate whether each material, system or method listed is relevant to your study. If you are not sure if a list item applies to your research, read the appropriate section before selecting a response.

Materials & experimental systems

n/a	Involved in the study
☒	☐ Antibodies
☒	☐ Eukaryotic cell lines
☒	☐ Palaeontology and archaeology
☒	☐ Animals and other organisms
☒	☐ Clinical data
☒	☐ Dual use research of concern
☒	☐ Plants

Methods

n/a	Involved in the study
☒	☐ ChIP-seq
☒	☐ Flow cytometry
☒	☐ MRI-based neuroimaging
