# Supplementary material for: Author Correction: Global climate-change trends detected in indicators of ocean ecology
Source: Nature. 2024 Oct 24;635(8037):E2. doi: 10.1038/s41586-024-08090-9 (PMC11541188; doi:10.1038/s41586-024-08090-9)
Supplement: Supplementary file 1 — Original and corrected figures [file 41586_2024_8090_MOESM1_ESM.pdf]

nature portfolio

https://doi.org/10.1038/s41586-024-08090-9

# Supplementary information

# Author Correction: Global climate-change trends detected in indicators of ocean ecology

In the format provided by the authors and unedited

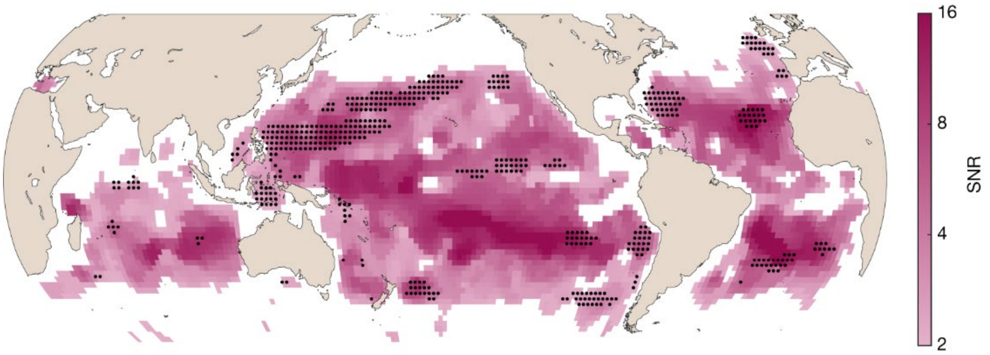


Original Fig. 1

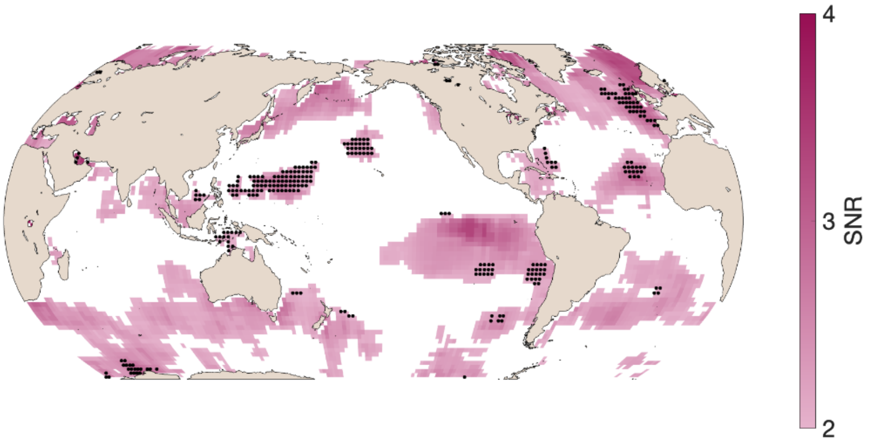


Corrected Fig. 1

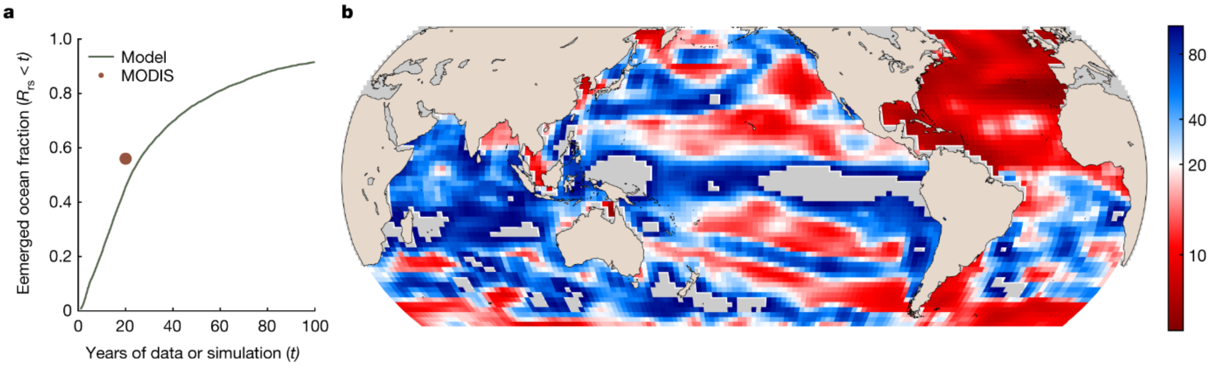


Original Fig. 2

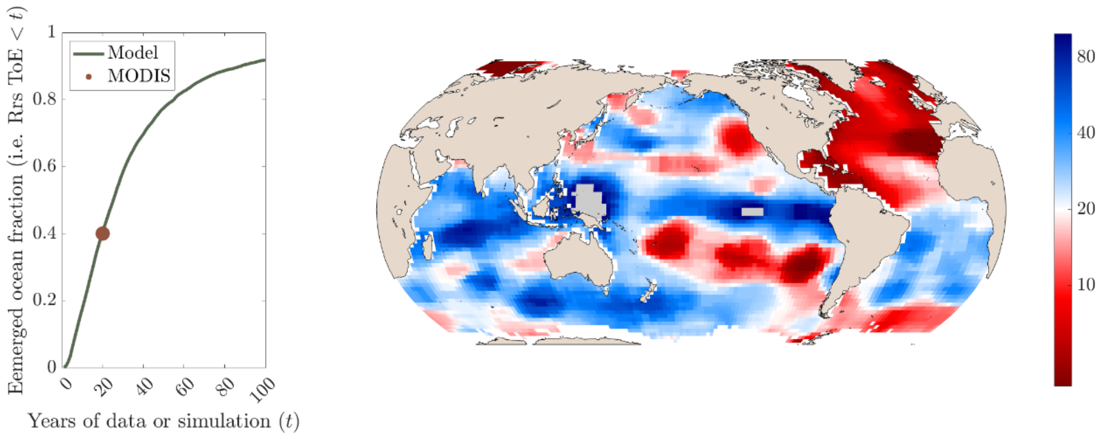


Corrected Fig. 2

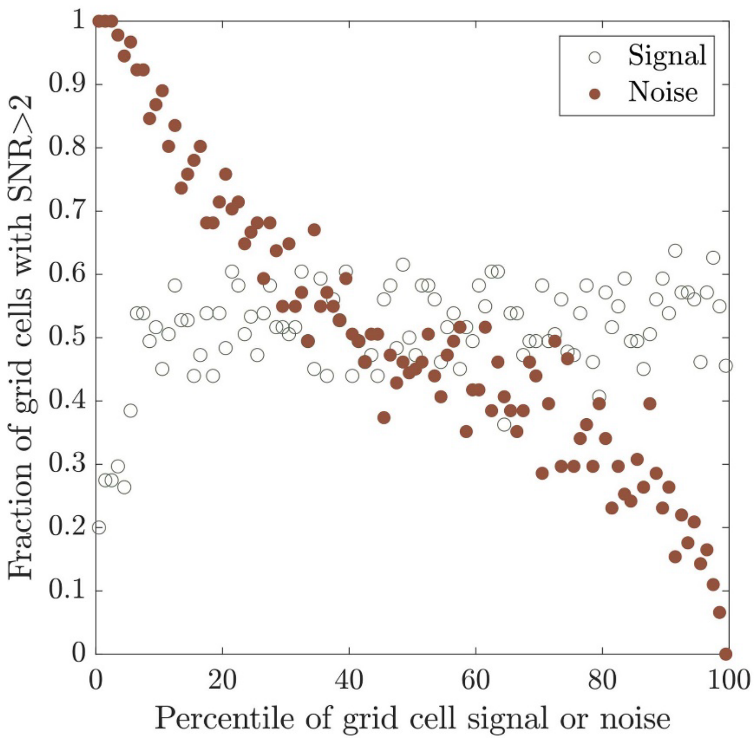


Original Extended Data Fig. 2

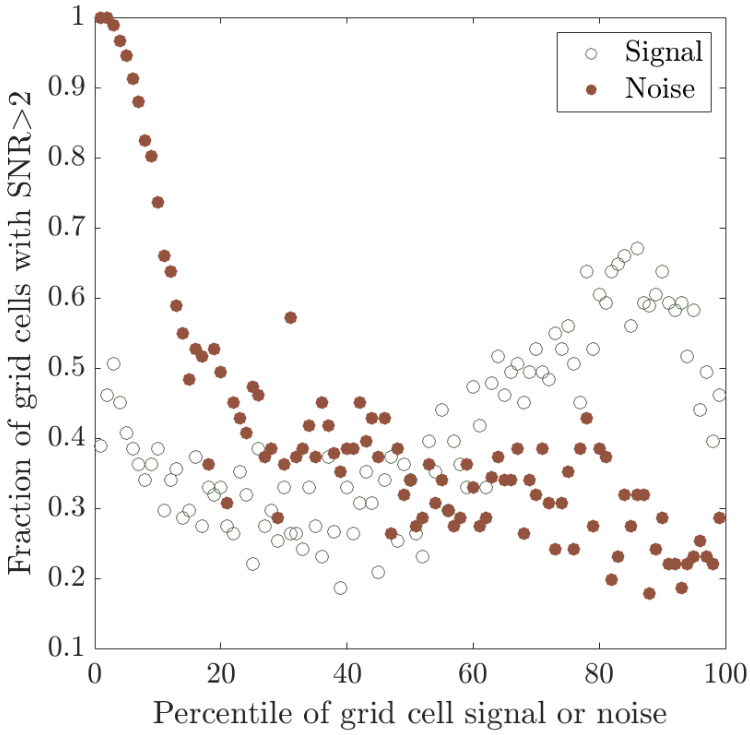


Corrected Extended Data Fig. 2

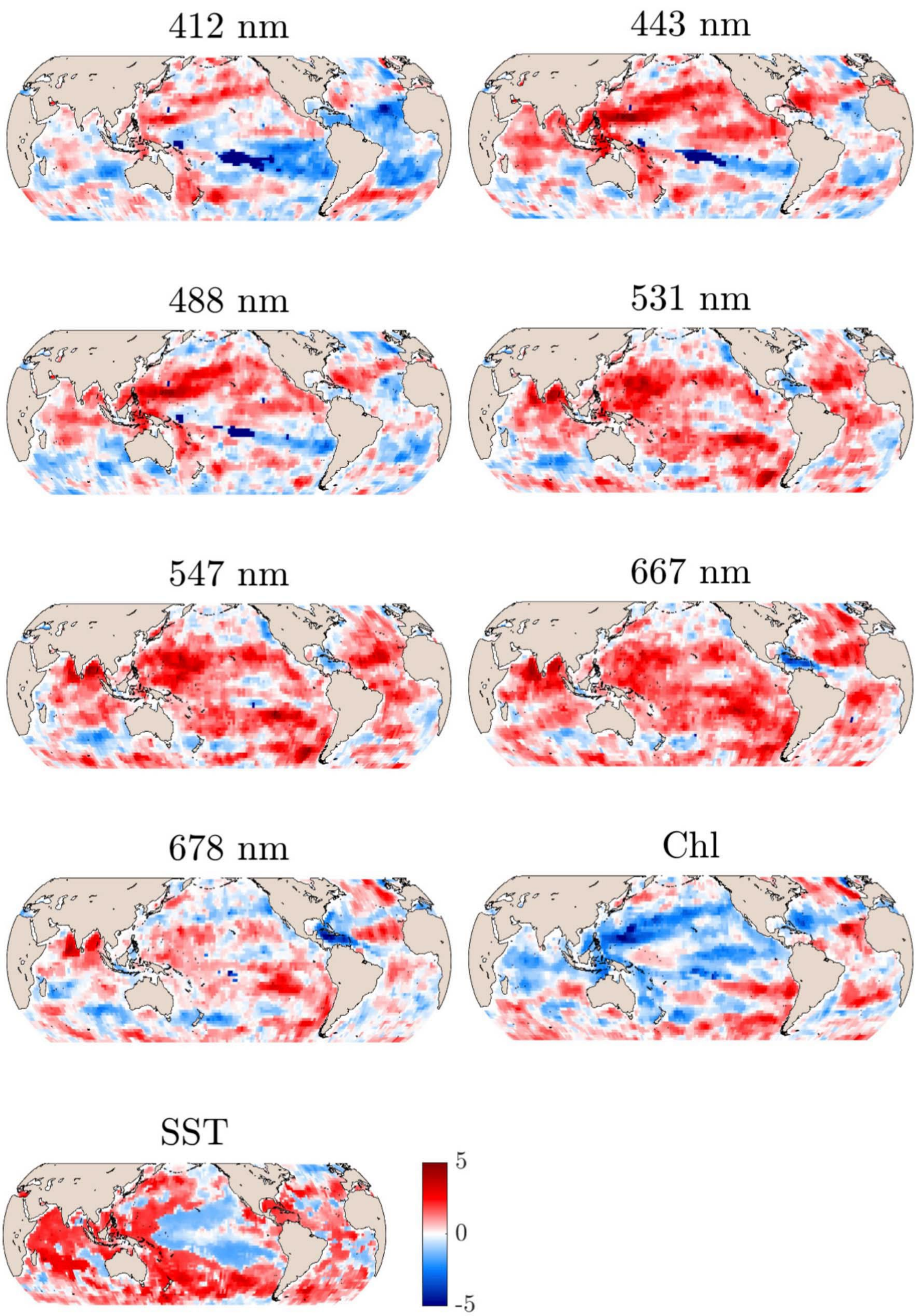


Original Extended Data Fig. 3

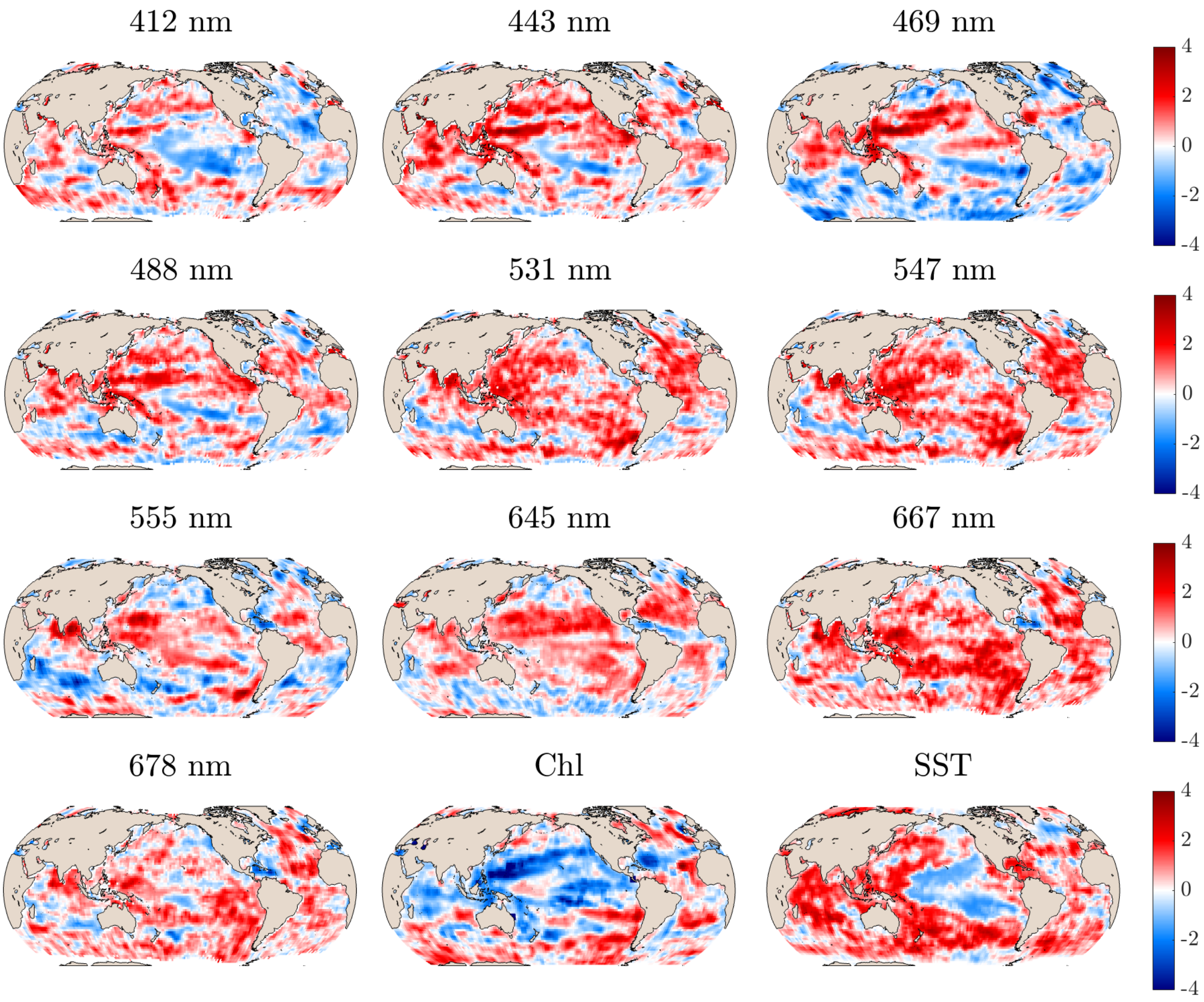


Corrected Extended Data Fig. 3

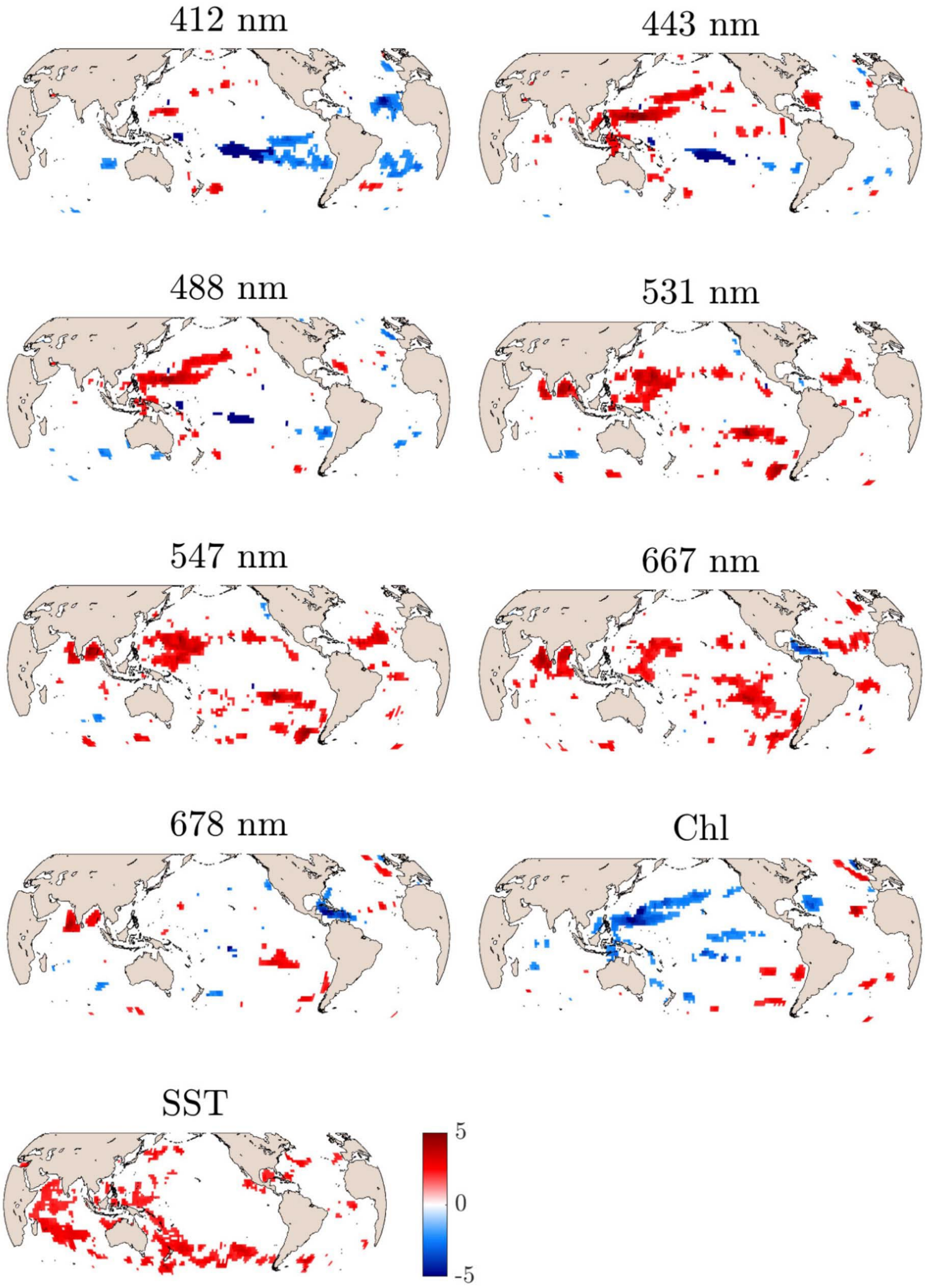


Original Extended Data Fig. 4

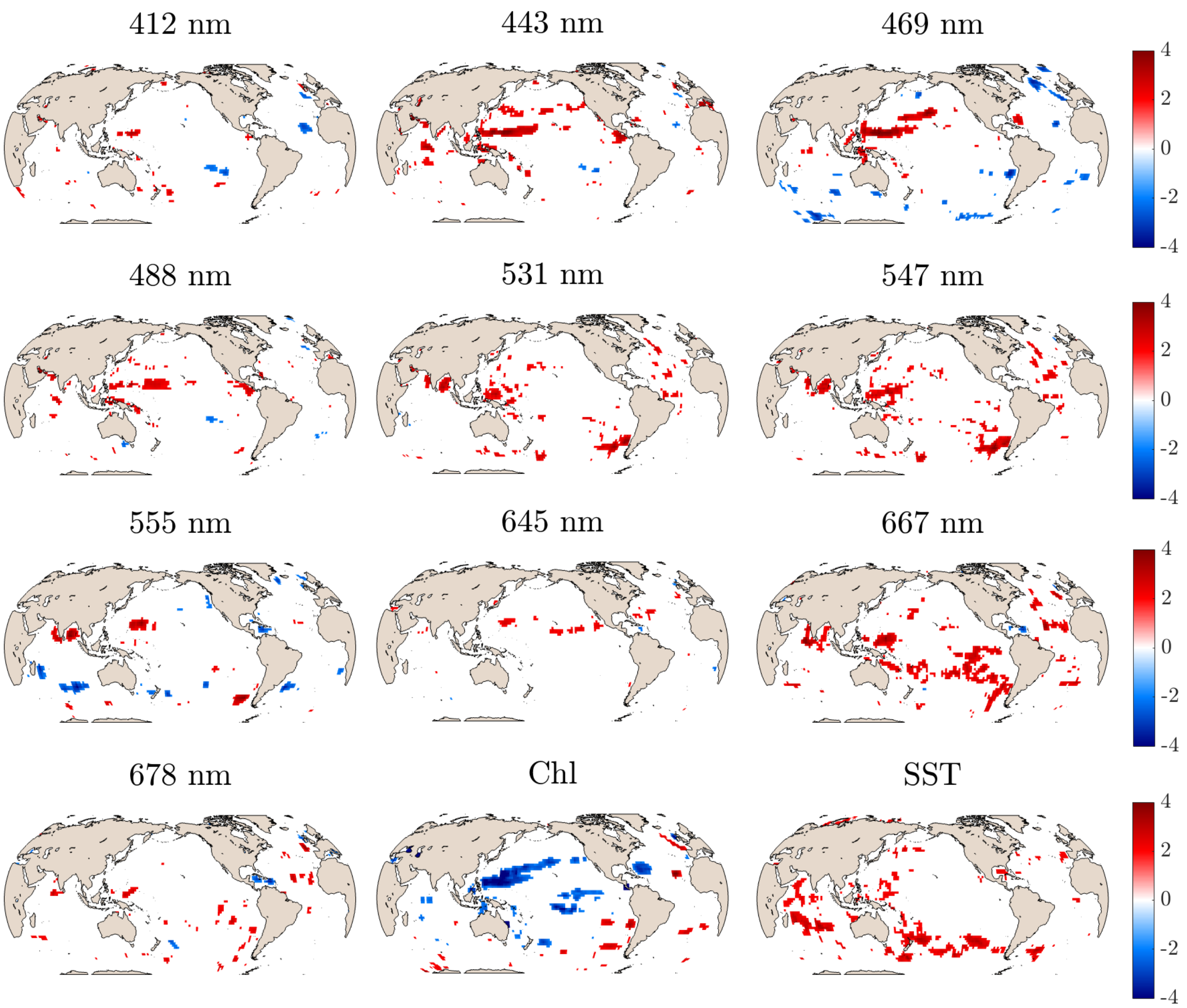

Corrected Extended Data Fig. 4
